# Complement inhibition prevents glial nodal membrane injury in a GM1 antibody-mediated mouse model

**DOI:** 10.1093/braincomms/fcac306

**Published:** 2022-11-23

**Authors:** Clare I Campbell, Rhona McGonigal, Jennifer A Barrie, Jolien Delaere, Laura Bracke, Madeleine E Cunningham, Denggao Yao, Tim Delahaye, Inge Van de Walle, Hugh J Willison

**Affiliations:** Institute of Infection, Immunity and Inflammation, University of Glasgow, Glasgow G12 8TA, UK; Institute of Infection, Immunity and Inflammation, University of Glasgow, Glasgow G12 8TA, UK; Institute of Infection, Immunity and Inflammation, University of Glasgow, Glasgow G12 8TA, UK; argenx BV, Zwijnaarde, 9052 Ghent, Belgium; argenx BV, Zwijnaarde, 9052 Ghent, Belgium; Institute of Infection, Immunity and Inflammation, University of Glasgow, Glasgow G12 8TA, UK; Institute of Infection, Immunity and Inflammation, University of Glasgow, Glasgow G12 8TA, UK; argenx BV, Zwijnaarde, 9052 Ghent, Belgium; argenx BV, Zwijnaarde, 9052 Ghent, Belgium; Institute of Infection, Immunity and Inflammation, University of Glasgow, Glasgow G12 8TA, UK

**Keywords:** Guillain–Barré syndrome, complement inhibition, glial membrane injury, paranodal loops, axo-glial junction

## Abstract

The involvement of the complement pathway in Guillain–Barré syndrome pathogenesis has been demonstrated in both patient biosamples and animal models. One proposed mechanism is that anti-ganglioside antibodies mediate neural membrane injury through the activation of complement and the formation of membrane attack complex pores, thereby allowing the uncontrolled influx of ions, including calcium, intracellularly. Calcium influx activates the calcium-dependent protease calpain, leading to the cleavage of neural cytoskeletal and transmembrane proteins and contributing to subsequent functional failure. Complement inhibition has been demonstrated to provide effective protection from injury in anti-ganglioside antibody-mediated mouse models of axonal variants of Guillain–Barré syndrome; however, the role of complement in the pathogenesis of demyelinating variants has yet to be established. Thus, it is currently unknown whether complement inhibition would be an effective therapeutic for Guillain–Barré syndrome patients with injuries to the Schwann cell membrane. To address this, we recently developed a mouse model whereby the Schwann cell membrane was selectively targeted with an anti-GM1 antibody resulting in significant disruption to the axo-glial junction and cytoplasmic paranodal loops, presenting as conduction block. Herein, we utilize this Schwann cell nodal membrane injury model to determine the relevance of inhibiting complement activation. We addressed the early complement component C2 as the therapeutic target within the complement cascade by using the anti-C2 humanized monoclonal antibody, ARGX-117. This anti-C2 antibody blocks the formation of C3 convertase, specifically inhibiting the classical and lectin complement pathways and preventing the production of downstream harmful anaphylatoxins (C3a and C5a) and membrane attack complexes. Here, we demonstrate that C2 inhibition significantly attenuates injury to paranodal proteins at the node of Ranvier and improves respiratory function in *ex vivo* and *in vivo* Schwann cell nodal membrane injury models. In parallel studies, C2 inhibition also protects axonal integrity in our well-established model of acute motor axonal neuropathy mediated by both mouse and human anti-GM1 antibodies. These data demonstrate that complement inhibition prevents injury in a Schwann cell nodal membrane injury model, which is representative of neuropathies associated with anti-GM1 antibodies, including Guillain–Barré syndrome and multifocal motor neuropathy. This outcome suggests that both the motor axonal and demyelinating variants of Guillain–Barré syndrome should be included in future complement inhibition clinical trials.

## Teaser

Campbell *et al.* demonstrate that complement inhibition at the C2 component prevents Schwann cell paranodal loop injury in a mouse model of Guillain–Barré syndrome. This outcome suggests that both axonal and demyelinating variants of Guillain–Barré syndrome could reasonably be included in future clinical trials of classical pathway complement inhibitors

## Graphical Abstract

**Graphical abstract:**
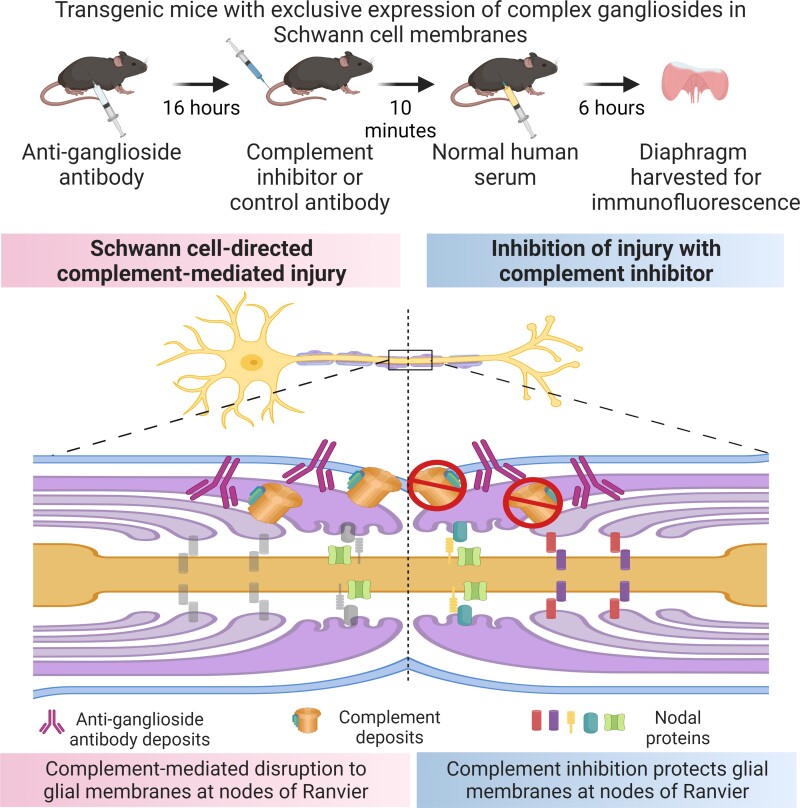

## Introduction

Guillain–Barré syndrome is a heterogeneous peripheral neuropathy associated with a wide range of pathological features. The principal subtype of Guillain–Barré syndrome across Europe and North America is acute inflammatory demyelinating polyneuropathy (AIDP)^1^ which is characterized by segmental demyelination with nerve conduction failure.^2^ Autopsy analysis from patients with AIDP has demonstrated the deposition of immunoglobulin and complement products on the outer surface of Schwann cells and the presence of macrophages adjacent to fibres undergoing extensive demyelination.^3,4^ Although the responsible antigenic target(s) on Schwann cell membranes have yet to be fully identified, AIDP pathogenesis is hypothesized to be mediated by the binding of autoantibodies, including many different anti-glycolipid and anti-ganglioside antibodies (AGAbs), that results in complement activation and subsequent membrane injury. Autoantibodies against GM1 ganglioside are strongly associated with acute motor axonal neuropathy (AMAN)^5^ and multifocal motor neuropathy, which has both axonal and demyelinating features.^6^ However, anti-GM1 antibodies are also found in some cases of AIDP.^7^ Our previous animal studies have demonstrated that the binding of AGAbs at distal motor nerve terminals activates the classical complement pathway, leading to the formation of membrane attack complex (MAC) pores in the membrane.^8^ These pores allow the bi-directional flow of ions and water, disrupting ionic homeostasis and culminating in swelling and cell lysis.^9^ A consequence of this is an intracellular calcium influx causing a retrograde calcium wave^10^ and the subsequent activation of the calcium-dependent protease calpain,^11,12^ known to cleave neural cytoskeletal structural proteins such as neurofilament, actin and ankyrin.^13,14^ This distal motor nerve injury results in distal axonal and neuromuscular transmission blocks, presenting as paralysis.^12,15^

The complement cascade is currently of significant interest as a therapeutic target for Guillain–Barré syndrome. Intervention at various stages of the complement pathway is now possible with emergent therapeutics.^16–18^ Halstead *et.al*.^15^ established that preventing the cleavage of C5 and the formation of terminal MAC pores attenuated injury in an *in vivo* model representative of Miller Fisher syndrome variant of Guillain–Barré syndrome. Later, it was demonstrated that inhibiting C1q, part of the complement initiation complex in the classical pathway, was neuroprotective in an *in vivo* AMAN mouse model.^19^ However, the involvement of complement in the pathogenesis of AIDP has yet to be demonstrated in animal models, and thus it is undetermined whether complement inhibition would be an effective therapeutic for Guillain–Barré syndrome patients with Schwann cell membrane injury.

We have recently developed an acute *in vivo* Schwann cell nodal membrane injury model in *GalNAc-T^−/−^-Tg(glial)* mice, mediated by an anti-GM1 antibody.^20^*GalNAc-T^−/−^-Tg(glial)* mice have restricted expression of complex gangliosides, including GM1, to glial membranes,^21^ allowing us to selectively target the Schwann cell membrane in absence of confounding axonal membrane injury. Anti-GM1 antibody-mediated injury to paranodal loops at the most distal node of Ranvier (NoR) in this transgenic mouse resulted in significant disruption to paranodal proteins and architecture following complement activation and MAC pore formation.^20^ This model will be referred to as Schwann cell nodal membrane injury and likely forms the early stages of segmental demyelination.

Herein, we first confirmed that inhibition of C2 would attenuate injury in our established *ex vivo* AMAN mouse model mediated by either mouse or human anti-GM1 antibody. Next, we assessed the protection of the Schwann cell membrane following inhibition of the classical complement pathway by blocking C2 in *ex vivo* and *in vivo* Schwann cell nodal membrane injury models.

## Materials and methods

### Mice

*GalNAc-T^−/−^-Tg(neuronal)* and *GalNAc-T^−/−^-Tg(glial)* mice have been described previously.^19,21^ Briefly, neuronal and glial mice only synthesize complex gangliosides (including GM1). All mice were bred through multiple generations onto a C57BL/6 background and crossed with fluorescent adult B6.Cg-Tg mice that expressed intracytosolic cyan fluorescent protein (CFP) in their peripheral motor and sensory axons [Thy1-CFP]. For *in vivo* experiments, a total of 11 mice (five male, six female), ranging from 10 to 18 g, were used at 4–5 weeks. For *ex vivo*, a total of 24 mice (13 male, 11 female), ranging from 10 to 18 g, were used at 4–6 weeks of age. No sex-related differences were found. Animals were killed with a rising concentration of CO_2_ and cervical dislocation was then performed to confirm death. All procedures were conducted in accordance with a licence approved and granted by the United Kingdom Home Office (POC6B3485).

### Antibodies and normal human serum

The mouse monoclonal IgG3 anti-GM1 ganglioside antibody, used in all experiments, was generated as described previously.^22,23^ The human monoclonal IgM anti-GM1 antibody, SM1, was cloned from peripheral blood lymphocytes from a patient with multifocal motor neuropathy and fused with a mouse myeloma cell line, previously described in detail.^24^ Bro-2 is a monoclonal IgG4 anti-C2 antibody that is analogous to the previously published monoclonal IgG1 humanized anti-C2 antibody, ARGX-117.^25^ The anti-C2 humanized antibodies that were used, referred to throughout as ‘C2 inhibitor’, bind to the S2 domain of C2, preventing the formation of C3 convertase and, thus, the cleavage of C3. Blocking C2 will inhibit progression through C3, eliminating the production of harmful anaphylatoxins C3a and C5a and preventing the formation of MAC pores. Bro-2 (used in *ex vivo* studies), ARGX-117 (used for *in vivo* studies) and the IgG4 and IgG1 isotype-matched control monoclonal antibodies (mAb) were supplied by argenx (Zwijnaarde, Belgium). Normal human serum (NHS), used as a source of human complement, was taken from a single donor and stored in aliquots at −80°C.

The following were used for immunofluorescence staining to identify neural proteins and complement products: α-bungarotoxin (BTx) Alexa Fluor 555-conjugated (Thermo Fisher Scientific Cat# B35451, Research Resource Identifier (RRID):AB_2617152; 1/500) and rat anti-myelin basic protein (MBP) monoclonal antibody (Bio-Rad Cat# MCA409S, RRID:AB_325004; 1/500) were used throughout to label nerve terminals and compact myelin, respectively; rabbit anti-human C1q complement fluorescein isothiocyanate conjugated polyclonal antibody (Agilent Cat# F0254, RRID:AB_2335713; 1/100); rabbit anti-human C3c complement fluorescein isothiocyanate conjugated polyclonal antibody (Agilent Cat# F0201, RRID:AB_2335709; 1/300); mouse anti-human C5b-9 monoclonal antibody (Agilent Cat# M0777, RRID:AB_2067162; 1/50); mouse purified anti-neurofilament heavy phosphorylated monoclonal antibody (NFH; BioLegend Cat# 801602, RRID:AB_2715851); mouse anti-sodium channel pan monoclonal antibody (Nav; Sigma-Aldrich Cat# S8809, RRID: AB_477552); mouse anti-Ankyrin B monoclonal antibody (AnkB; UC Davis/NIH NeuroMab Facility Cat# N105/13, RRID: AB_2877371; 1/800 sections or 1/200 whole-mount); rabbit anti-CASPR1 polyclonal antibody (gifted by Professor E. Peles, Weizmann Institute, Rehovot, Israel; 1/1000 sections or 1/500 whole-mount) and rabbit anti-neurofascin pan polyclonal antibody (pan-NFasc; gifted by Professor P. Brophy, University of Edinburgh, Edinburgh, UK; 1/750). The secondary antibodies used are as follows: goat anti-rabbit IgG Alexa Fluor 555- or 647-conjugated antibody (Thermo Fisher Scientific Cat# A-21429, RRID: AB_2535850 or Thermo Fisher Scientific Cat# A-21446, RRID: AB_2535863; 1/500), goat anti-mouse IgG2a Alexa Fluor 488- (Thermo Fisher Scientific Cat# A-21131, RRID: AB_2535771; 1/500) or 647- conjugated antibody (Thermo Fisher Scientific Cat# A-21241, RRID: AB_2535810; 1/500), goat anti-mouse IgG1 Alexa Fluor 647 (Thermo Fisher Scientific Cat# A-21240, RRID: AB_2535809), goat anti-mouse IgG3 Alexa Fluor 488 (Southern Biotech Cat# 1101–30, RRID: AB_2895720; 1/300) and goat anti-human IgM Alexa Fluor 488 (Thermo Fisher Scientific Cat# A-21215, RRID: AB_2535800; 1/500).

### *Ex vivo* C2 complement inhibition studies

Triangularis sterni (TS) muscle was dissected and mounted in oxygenated Ringer’s solution (116 mM NaCl, 4.5 mM KCl, 1 mM MgCl_2_, 1 mM NaH_2_PO_4_, 23 mM NaHCO_3_, 11 mM glucose, 2 mM CaCl_2_, made up in dH_2_O, pH 7.3) as described previously.^11^ The *ex vivo* model used was adapted from McGonigal and colleagues.^20^ When assessing the integrity of the nerve terminal in *GalNAc-T^−/−^-Tg(neuronal)* mice, working solutions of 100 μg/mL Bro-2 (or 100 μg/mL IgG4 control mAb) and 40% NHS were prepared in Ringer’s solution. Solutions were left for 10 minutes before adding 100 μg/mL anti-GM1 mAb. Control tissue received 40% NHS-only in Ringer’s solution. Working solutions were added to the TS from *GalNAc-T^−/−^-Tg(neuronal)* mice for 1 hour at 32°C in a humidifying chamber. Following antibody and NHS incubation, the TS was washed in cold Ringer’s solution prior to being fixed in 4% paraformaldehyde for 20 minutes at 4°C. The TS was then washed for 10 minutes in 1× phosphate buffered saline (PBS), 0.1 M glycine, followed by 1xPBS again at room temperature. The muscle was carefully cut away from the intercostal muscles, snap frozen and stored at −80°C until required for immunofluorescence staining.

To investigate the integrity of the axo-glial junction at the distal NoR, the same method was used as described above, except 200 μg/mL Bro-2 or IgG4 control mAb was used, and working solutions were added to the TS from both *GalNAc-T^−/−^-Tg(neuronal)* and *GalNAc-T^−/−^-Tg(glial)* mice and left to incubate for 4 hours, as determined previously,^11^ at 32°C in a humidifying chamber. The TS was then washed and fixed as described above.

For human anti-GM1 antibody *ex vivo* studies, TS from *GalNAc-T^−/−^-Tg(neuronal)* mice were dissected and mounted in Ringer’s solution. To optimize binding of the SM1 antibody, the TS was then incubated in neuraminidase (10 u/mL) for 1 hour at 32°C.^26^ Following washes in cold Ringer’s solution, the TS was incubated in 75 μg/mL SM1 for 90 minutes at 32°C followed by 30 minutes at 4°C. Solutions of 100 μg/mL Bro-2 (or 100 μg/mL IgG4 control mAb) and 40% NHS were prepared in Ringer’s solution and were left for 10 minutes. The TS was washed in cold Ringer’s solution before being incubated in C2 inhibitor and NHS for 1 hour at room temperature. The TS was then washed and fixed as described previously.

### *In vivo* C2 complement inhibition studies

The *in vivo* model used here was modified from McGonigal *et al*.^20^ Due to the proximity of the diaphragm to the site of injection, this results in paralysis of the intra-diaphragmatic distal phrenic nerve and can be identified by a reduction in tidal volume, measured by whole-body plethysmography (WBP).^15^ Mice were habituated to the WBP chambers (Electro-Medical Measurement Systems, Hants, UK) for 1 hour. The following day, a 20-minute baseline recording was taken before mice were injected intraperitoneally (IP) with 50 mg/kg anti-GM1 mAb. The next morning, mice received 200 mg/kg ARGX-117 or 200 mg/kg IgG1 control mAb, administered intravenously. After 10 minutes, 30 μL/g NHS was delivered IP as a source of complement. Plethysmography was performed at 5-hours post NHS injection (post-injury). Littermates were used as naïve controls, receiving 1xPBS instead of antibody and NHS injections. At 6 hours post-injury, mice were asphyxiated with a rising concentration of CO_2_, followed by a secondary measure. Blood samples and diaphragms were harvested. The blood was centrifuged at 10 000 g for 10 minutes at 4°C, the supernatant was collected and stored at −80°C until use. Half of the diaphragm was snap frozen and stored at −80°C, the remaining half was fixed in 4% paraformaldehyde for 1 hour at 4°C before being washed for 10 minutes in 1× PBS, 0.1 M glycine, followed by 1×PBS again at room temperature. The fixed diaphragm was cryoprotected in 30% sucrose overnight at 4°C then stored at −80°C until required for immunofluorescence staining.

### ELISA

An ELISA was performed to confirm the presence of anti-GM1 mAb in the mouse sera from the *in vivo* experiments, as described in detail previously.^26^ Sera from mice was applied at 1/50 dilution.

### Complement C2 assay

An automated complement C2 assay was performed by argenx using a Gyrolab xPlore^TM^ Single CD workstation and Gyrolab Bioaffy 200 CD (Gyrolab Protein Technologies, Cat. P0004180), to assess levels of human C2 unbound by ARGX-117 (further referred to as ‘free C2’) in the mouse sera from the *in vivo* experiment. An initial needle and double column rinse were performed with wash buffer (1x PBS with 0.01% Tween-20; Sigma-Aldrich, Cat# P1379 and 0.02% sodium azide; Merck, Cat# 106688). The biotinylated capture molecule ARGX-117 (50 μg/mL in 1xPBS-T (1xPBS with 0.05% Tween-20)) was introduced into the microstructure of the Gyrolab Bioaffy CD to bind the streptavidin-coated beads located in small capillaries. The columns were then washed in 1xPBS-T prior to the addition of known dilutions of recombinant human C2 (Calbiochem/Merck, Cat# 204882) and unknown serum samples (1:3 diluted) into the microstructures. Samples were prepared in Rexxip HX buffer (Gyros Protein Technologies, Cat# P0020033) supplemented with 1.25 mM CaCl_2_ (Sigma, Cat# 21115). Any human free C2 present in mouse sera was captured by the biotinylated ARGX-117 on the affinity capture column. The columns were washed in 1xPBS-T before Alexa Fluor 647-labelled mAb32 (3 μg/mL in Rexxip F buffer (Gyros Protein Technologies; Cat# P0004825)) was added to detect human free C2 captured by the biotinylated ARGX-117. After a final wash in 1×PBS-T, binding of Alexa Fluor 647-labelled mAb32 in each capillary column was assessed by a laser-induced signal (photomultiplier tube setting 1%) generating an obtained signal measured as responsive units. The obtained signals were used to estimate human free C2 levels in the unknown samples by comparing them against a standard curve (five parameter logistic fit, 1/y^2^ weighting).

### Immunofluorescence staining

Whole-mount TS preparations were used for immunofluorescence analysis following *ex vivo* injury with anti-GM1 mAb and NHS. The TS was halved post-fixation so that two-marker studies could be performed per mouse. The diaphragm (previously exposed to anti-GM1 mAb and NHS *in vivo*) was sectioned at 10 μm and collected onto 3-aminopropyltriethoxysilane coated slides. For each mouse, each marker was stained in duplicate. TS and diaphragm were permeabilized in 100% EtOH for 10 minutes at −20°C and then washed thoroughly in 1x PBS. To assess Nav clusters at the node, tissue was blocked in 10% blocking solution (normal goat serum in 1x PBS) for 1 hour at 4°C prior to antibody incubations. Primary antibody solutions were made up in 0.1% Triton X-100 and 3% blocking solution and incubated overnight at 4°C. The tissue was washed in 1xPBS and then incubated in a secondary antibody solution, made up of 1x PBS and 3% blocking solution, for 2 hours at room temperature. The tissue was washed in 1x PBS and mounted in Citifluor.

### Image acquisition and quantification

All imaging was performed using a Zeiss Axio Z1 Imager with an Apotome attachment (removed for imaging sections, attached for imaging whole-mount tissue) and captured with Zen Blue Edition software (version 6.1.7). Each stain was performed in duplicate for each mouse and all tissue was coded prior to imaging. Every distal nerve identified by MBP and BTx staining was imaged in each diaphragm/TS. Z-stacks with a 0.4 μm interval were taken using a 63 × oil objective. For these images, 24–89 nerve terminals and 5–44 distal nerves were analysed per mouse. The distal nerve was defined as the nerve terminal, first internode and first NoR identified by a gap in MBP staining. When assessing complement deposition, if deposits were detected overlying the distal internode and/or NoR, this was scored as positive. To assess the integrity of proteins at the NoR, presence/absence analysis was performed. When assessing the integrity of paranodal markers AnkB, CASPR1 and NF155, presence was defined as both domains and absence was defined as a single domain or complete loss of staining.

### Experimental design

For *in vivo* experiments, mice were randomly assigned to treatment groups using an online random team generator. Confounding factors were controlled by administering repeated treatments to the mice in the same order and using tunnel handling to minimize handling stress. Exclusion criteria were determined *a priori.* Animals were excluded if anti-GM1 mAb was absent from the serum or if complement was not activated, as determined by negative C1q staining. One mouse in the C2 inhibitor group from the *in vivo* experiment was removed from all analysis due to an absence of C1q staining. The tissue was coded by an independent individual prior to immunofluorescence analysis and the user was blinded until the analysis was completed.

A power analysis was performed using G*Power software (version 3.0.10). The required sample size for 80% power assuming a 0.05 significance level was determined to be *n* = 3 for each treatment group.

### Statistical analysis

All statistics were performed using GraphPad Prism 6, assuming a significance level of 0.05. To display presence/absence, scatter plots with bars were used. The error bars represent the standard error of the mean (SEM) for each treatment group. Data was normally distributed as determined by a Shapiro–Wilk test and so a one-way ANOVA was performed to test for significant differences between treatment groups.

## Results

### C2 inhibition attenuates injury in an *ex vivo* AMAN mouse model

**Figure 1:**
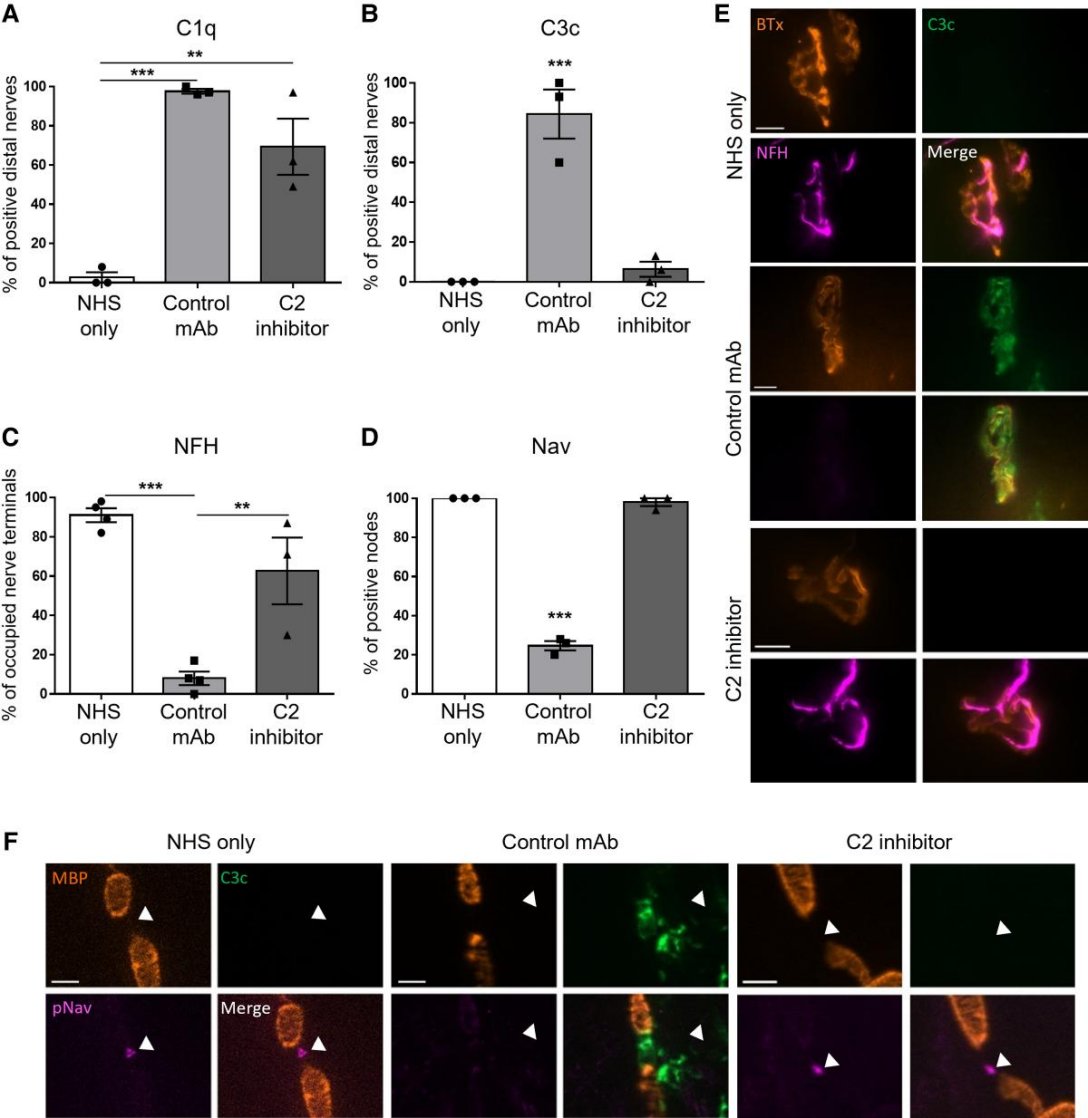
**C2 inhibition in an *ex vivo* mouse anti-GM1 mAb-mediated AMAN model.** TS muscle from *GalNAc-T^−/−^-Tg(neuronal)* mice was incubated in 100 μg/mL anti-GM1 mAb, 100 μg/mL of C2 inhibitor or control mAb and 40% NHS for 1 hour at 32°C. The NHS-only control mice received PBS and NHS. (**A**) C1q deposits were detected at significantly more distal nerves in the control mAb (*P* < 0.001) and C2 inhibitor group (*P* < 0.01) compared with NHS-only. (**B**) C3c deposits were detected in the control mAb group but not in the NHS-only or C2 inhibitor groups (*P* < 0.001), *n* = 3/treatments. (**C** and **D**) Axonal integrity was assessed at the nerve terminal and distal NoR by staining for the axonal structural proteins NFH and Nav clusters, respectively. (**C**) There was a significant loss of NFH staining overlying the nerve terminal in the control mAb group compared with all other treatment groups (*P* < 0.001 compared with NHS-only; *P* < 0.01 compared with C2 inhibitor). *n* = 4 NHS-only and control mAb; *n* = 3 C2 inhibitor. (**D**) Nav clusters were significantly disrupted in the control mAb group (*P* < 0.001) but were protected to comparable levels to the NHS-only control in the C2 inhibitor treatment group. *n* = 3/treatments. (**E**) NFH staining was protected in the C2 inhibitor group. Representative images demonstrate C3c deposits overlying the nerve terminal (identified by BTx) and the consequent loss of NFH staining in the control mAb group. C3c is absent from the NHS-only and C2 inhibitor groups and NFH is present overlying the nerve terminal. Scale bar = 10 μm. (**F**) Illustrative images show Nav clusters (indicated by arrowheads) at the node (identified by a gap in MBP staining) in NHS-only and C2 inhibitor groups. C3c deposits are detected at the node in the control mAb group, accompanied by a loss of Nav clusters. Scale bar = 5 μm. Results represented as average ± SEM. Each datapoint represents an individual animal. Statistical significance is assessed by performing a one-way ANOVA.

The effects of human C2 complement inhibition were first evaluated in our established axonal *ex vivo* injury model in *GalNAc-T^−/−^-Tg(neuronal)* mice^19,20^ to determine the efficacy of the C2 inhibitor. We first determined that the classical complement pathway had been activated by anti-GM1 mAb by assessing the presence of C1q, a component of the initiation complex (Fig. 1A). Then, to assess whether the classical complement pathway had been blocked at the level of C2, the downstream complement component C3c was studied (Fig. 1B, E and F). C1q and C3c complement deposits were not detected in nerve-muscle preparations that were incubated with NHS-only. This was expected as the NHS-only control was not incubated in anti-GM1 mAb. However, the classical complement pathway had been activated by anti-GM1 mAb, as demonstrated by C1q deposits detected in both the control mAb and C2 inhibitor groups. C3c deposits were only detected at the distal nerve in the control mAb group, indicating that the progression of the classical complement pathway beyond C1q had been blocked in the C2 inhibitor treatment group.

The neuroprotective effect of complement inhibition at C2 was then assessed by investigating axonal integrity at the nerve terminal and distal NoR by immunostaining for NFH and Nav, respectively (Fig. 1C–F). Nerve-muscle preparations treated with the control mAb presented a significant reduction of NFH-occupied nerve terminals (Fig.1C and E) and NoR with Nav clusters (Fig. 1D and F). In comparison, inhibition of C2 attenuated injury at the nerve terminal and distal NoR, affording protection to NFH and Nav immunofluorescence staining at comparable levels to the NHS-only control (Fig. 1E and F).

**Figure 2:**
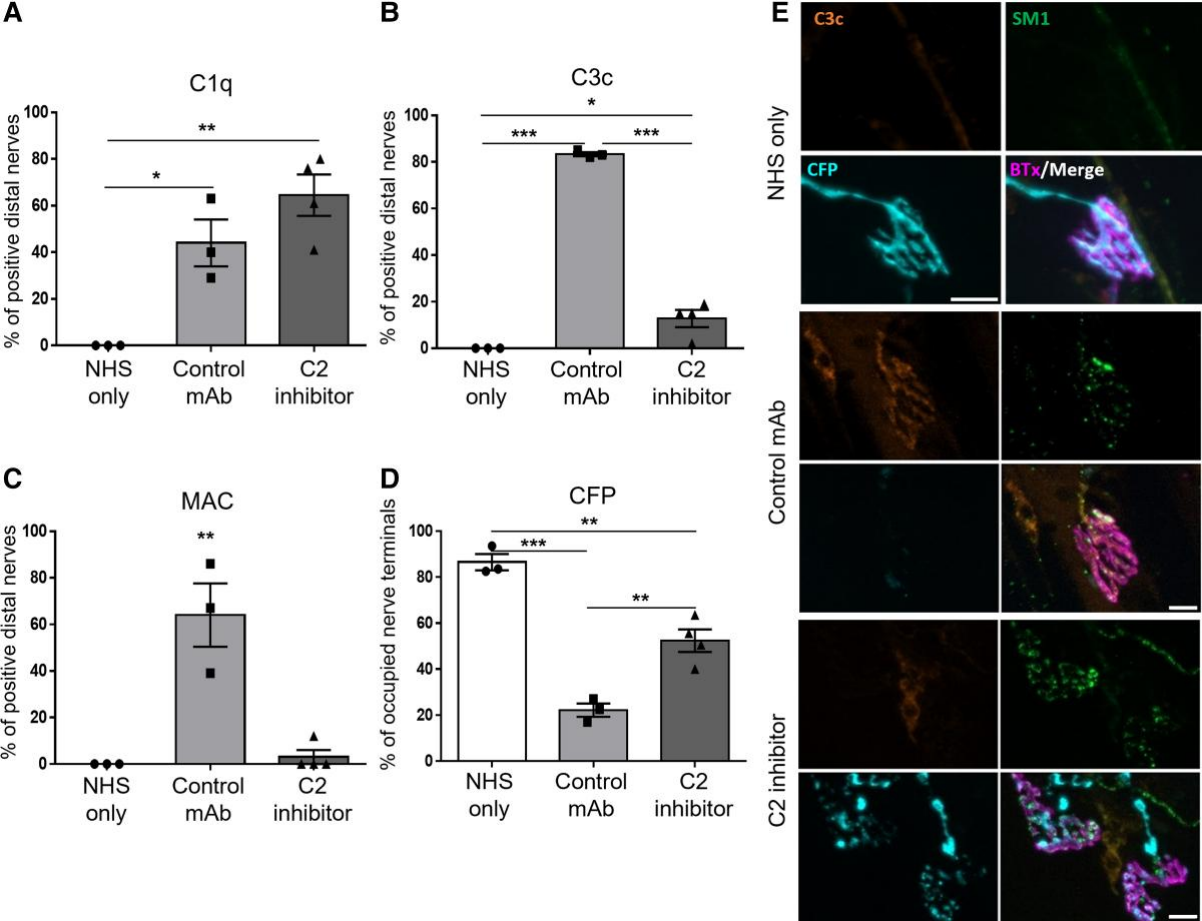
**Complement inhibition in an *ex vivo* AMAN mouse model mediated by a human anti-GM1 antibody.** TS muscle from *GalNAc-T^−/−^-Tg(neuronal)* mice was incubated in 10 u/mL neuraminidase for 1 hour at 32°C, then in 75 μg/mL SM1 and 100 μg/mL of C2 inhibitor or control mAb for 90 minutes at 32°C and 30 minutes at 4°C. TS was then incubated in 40% NHS for 1 hour at room temperature. (**A–C**) Complement products (C1q, C3c and MAC) were assessed to confirm complement activation and subsequent blockade of the pathway. (**A**) C1q deposits were detected at significantly more distal nerves in the control mAb (*P* < 0.05) and C2 inhibitor groups (*P* < 0.01) compared with the NHS-only group. (**B**) C3c and (**C**) MAC deposits were detected at significantly more distal nerves in the control mAb group compared with all other treatment groups (*P* < 0.001 C3c; *P* < 0.01 MAC). There were significantly more C3c deposits detected in the C2 inhibitor group (*P* < 0.05) compared with the NHS-only group but the percentage of distal nerves with MAC deposits was comparable between the C2 inhibitor and NHS-only groups. (**D**) Axonal integrity was investigated indirectly by assessing the presence of intracytosolic CFP at the nerve terminal. CFP was significantly reduced in the control mAb group compared with the NHS-only group (*P* < 0.001). Although there was a significant loss of CFP in the C2 inhibitor group in comparison to the NHS-only group (*P* < 0.01), CFP was significantly protected in comparison to the control mAb (*P* < 0.01). (**E**) Representative images demonstrate C3c deposits and SM1 deposits overlying the nerve terminal (identified by BTx) and the consequent loss of CFP staining in the control mAb group. C3c is absent from the NHS-only and C2 inhibitor groups and CFP is present overlying the nerve terminal, despite SM1 deposits at the nerve terminal in the C2 inhibitor group. Scale bar = 10 μm. *n* = 4 C2 inhibitor; *n* = 3 NHS-only and control mAb. Results represented as average ± SEM. Each data-point represents an individual animal. A one-way ANOVA was performed to test for statistical significance.

We next investigated the effects of C2 inhibition in our *ex vivo* AMAN mouse model, mediated by a human anti-GM1 IgM antibody (SM1). The classical complement pathway was activated by SM1 in both the control mAb and C2 inhibitor groups as demonstrated by the presence of C1q deposits (Fig. 2A). C3c and MAC complement deposits were present at a significantly higher number of nerve terminals in the control mAb group in comparison to the C2 inhibitor group, signifying that the classical complement pathway had been blocked by the C2 inhibitor (Fig. 2B and C). As expected, no complement products were detected in the NHS-only group. It has previously been demonstrated that CFP is lost through MAC pores in the membrane in our injury model^11^ and thus, the presence of CFP can be used indirectly to assess axonal integrity following complement inhibition (Fig. 2D and E). There was a significant loss of CFP immunofluorescence in nerve-muscle preparations treated with control mAb in comparison to the NHS-only group. Despite the significant loss of CFP in the C2 inhibitor group in comparison to the NHS-only group, C2 inhibition offered significant protection to CFP in comparison to the control mAb.

In summary, inhibition of C2 protects axonal integrity at the nerve terminal and distal NoR following mouse and human anti-GM1-mediated injury to the axonal membrane in this *ex vivo* injury model.

### Inhibition of C2 offers protection to the axo-glial junction in an *ex vivo* Schwann cell nodal membrane injury model

Having demonstrated the neuroprotective effects of C2 inhibition in the *ex vivo* axonal injury model, the therapeutic effects of classical complement inhibition were assessed in our *ex vivo* anti-GM1 mAb-mediated Schwann cell nodal membrane injury model in *GalNAc-T^−/−^-Tg(glial)* mice.

**Figure 3:**
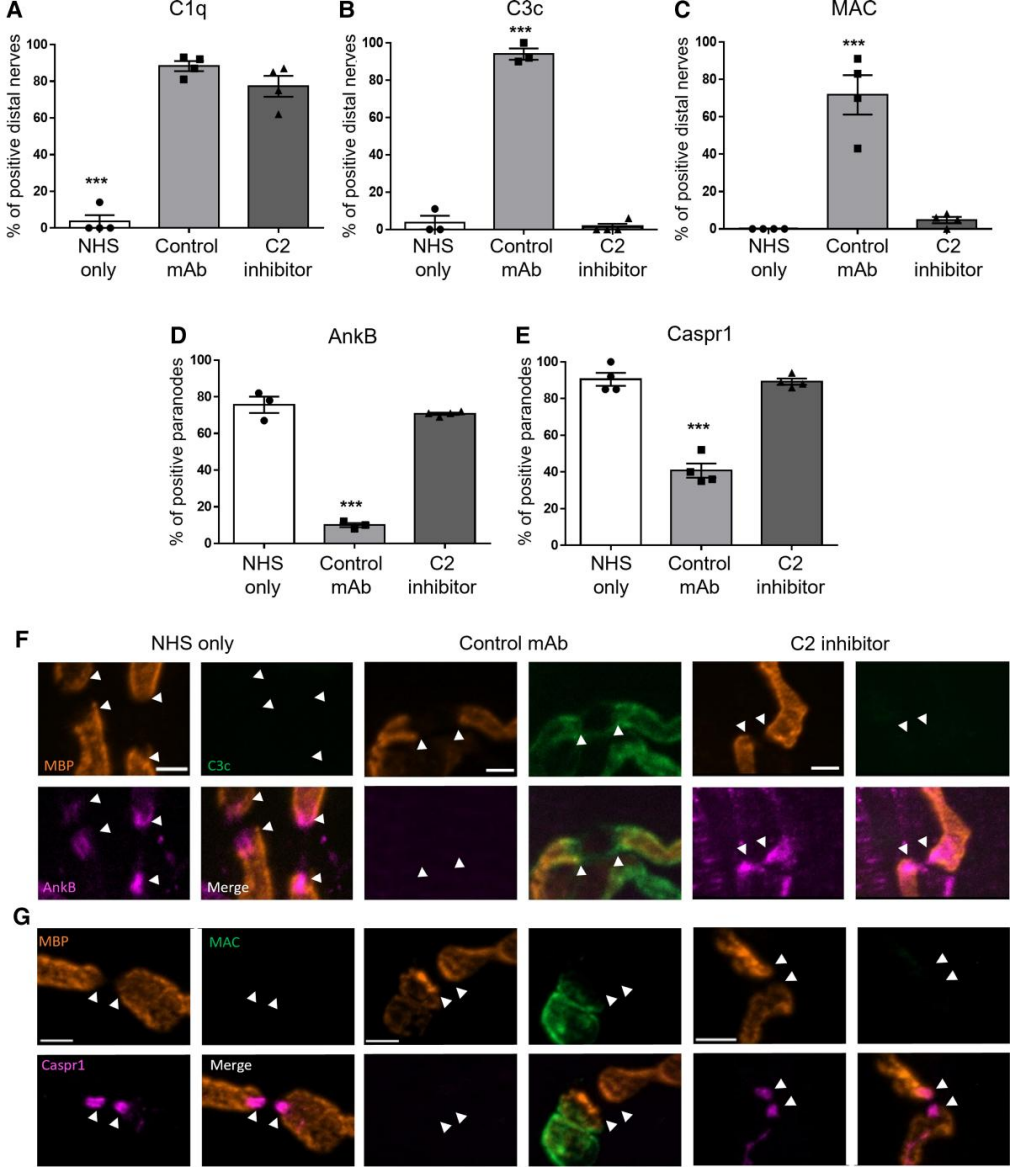
**Effects of C2 inhibition in an *ex vivo* Schwann cell nodal membrane injury model.** TS muscle from *GalNAc-T^−/−^-Tg(glial)* mice was incubated in 100 μg/mL anti-GM1 mAb, 200 μg/mL C2 inhibitor or control mAb and 40% NHS for 4 hours at 32°C. (**A–C**) Complement products (C1q, C3c and MAC) were assessed to confirm complement activation and subsequent blockade of the classical pathway beyond C2. (**A**) C1q deposits were detected at significantly more distal nerves in the control mAb and C2 inhibitor groups compared with NHS-only (*P* < 0.001). (**B**) C3c and (**C**) MAC deposits were detected at significantly more distal nerves in the control mAb group compared with the NHS-only and C2 inhibitor groups (*P* < 0.001). C1q and MAC *n* = 4/treatment; C3c *n* = 3 NHS-only and isotype control, *n* = 4 C2 inhibitor. (**D**) The presence of the glial cytoskeletal protein AnkB was significantly reduced in the control mAb group compared with all other treatment groups (*P* < 0.001). *n* = 3 NHS-only and control mAb, *n* = 4 C2 inhibitor. (**E**) CASPR1 was significantly protected in the C2 inhibitor group compared with the control mAb group (*P* < 0.001). *n* = 4/treatment. (**F**) Representative images show AnkB present in NHS-only and C2 inhibitor groups. C3c deposits are present at the paranode (arrowheads) in the control mAb group with a loss of AnkB staining. (**G**) Illustrative images demonstrate CASPR1 being absent from the paranode (arrowheads) in the control mAb group and MAC deposits overlying the MBP staining. In contrast, no complement deposits are detected in the NHS-only and C2 inhibitor groups, but CASPR1 is present at the paranode. Scale bar = 5 μm. Results represented as average ± SEM. Each data-point represents an individual animal. Statistical significance is determined by performing a one-way ANOVA.

We first confirmed that the classical complement pathway had been activated by studying C1q. This was followed by studying downstream complement components, C3c and MAC, to determine whether the progression of the classical complement pathway had been blocked beyond C2. In the control mAb group, anti-GM1 mAb activated complement and resulted in C1q, C3c and MAC deposition along the distal nerve (Fig. 3A–C and F–G). C1q deposits were detected along the distal nerve following treatment with the C2 inhibitor, signifying activation of the classical complement pathway (Fig. 3A). However, C3c and MAC deposits were not detected in the C2 inhibitor group, confirming that the progression of the classical complement pathway was inhibited (Fig. 3B–C and F–G). As expected, no complement deposits were present in the NHS-only group. It has previously been demonstrated that MAC pore formation in the glial membrane significantly disrupts cytoskeletal and axo-glial proteins on the axonal and glial membranes at the paranode.^20^ Corresponding to previous findings, we demonstrate that the formation of MAC pores in the glial membrane in the control mAb group resulted in significant disruption to the glial cytoskeletal protein, AnkB and axonal cell adhesion molecule CASPR1, both located at the paranode (Fig. 3D–E). Blockade of the classical complement pathway at C2 protected the integrity of the paranodes, as demonstrated by the presence of AnkB and CASPR1 immunofluorescence staining, rescuing the phenotype to comparable levels as the NHS-only control (Fig. 3F–G).

These results demonstrate that C2 inhibition prevents anti-GM1 mAb-mediated disruption to the paranode in an *ex vivo* Schwann cell nodal membrane injury model.

### C2 inhibition protects the integrity of the axo-glial junction in an *in vivo* Schwann cell nodal membrane injury model

Following the demonstration of the protective effects of C2 inhibition in our *ex vivo* Schwann cell nodal membrane injury model, the effects of C2 inhibition were next investigated *in vivo*. Mice were passively immunized with anti-GM1 mAb followed 16 hours later with 200 mg/kg C2 inhibitor or control mAb, administered intravenously. NHS was then administered IP 10 minutes later as a source of complement. Littermates received PBS only and were used as naïve controls. Respiratory function was then monitored using WBP and the diaphragm was subsequently removed for immunofluorescence analysis.

**Figure 4:**
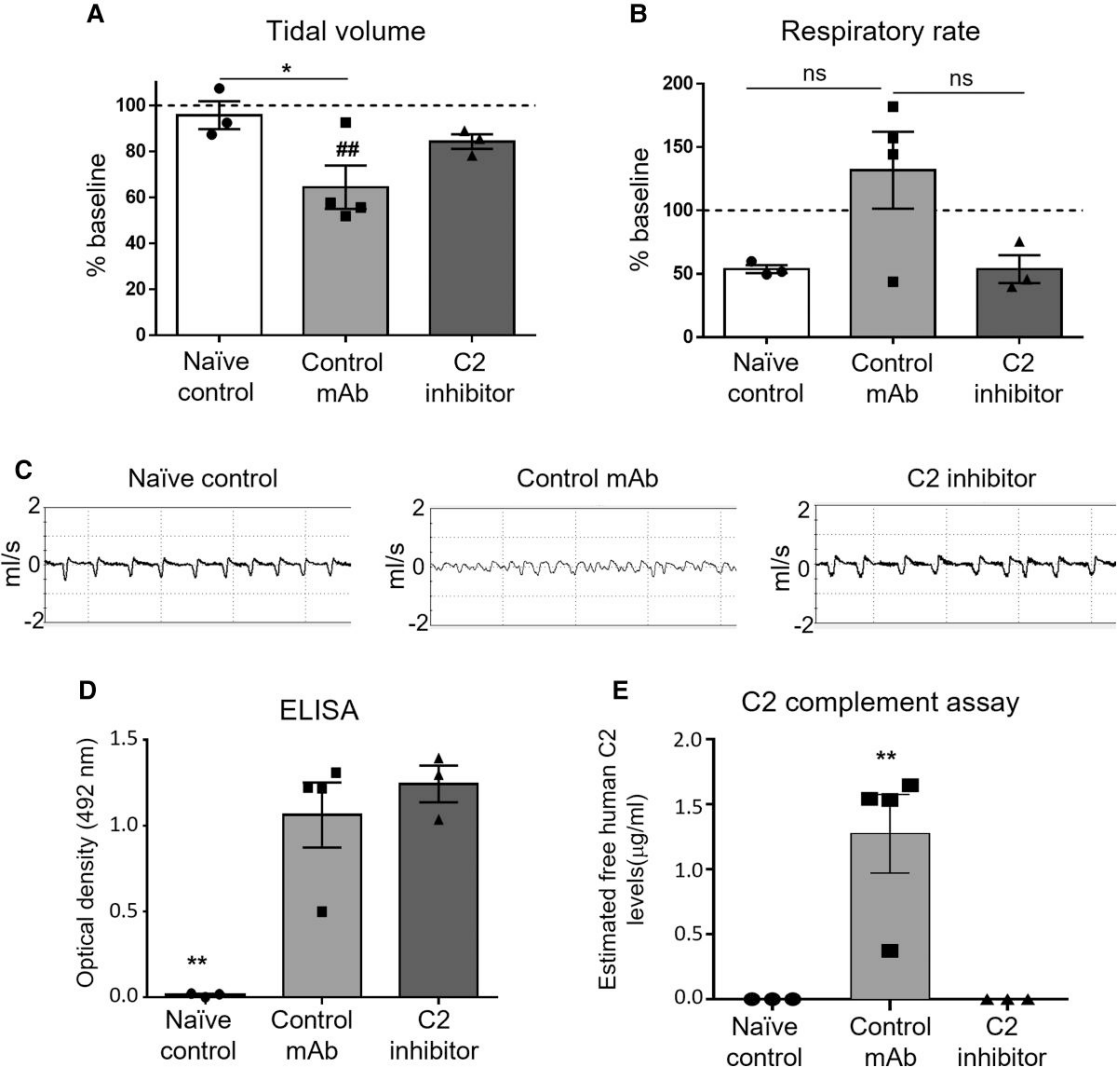
**C2 inhibition attenuates functional injury in an *in vivo* Schwann cell nodal membrane injury model.** A baseline WBP recording was taken prior to *GalNAc-T^−/−^-Tg(glial)* mice receiving 50 mg/kg anti-GM1 mAb IP. The following morning, mice received an intravenous injection of 200 mg/kg of C2 inhibitor or control mAb, followed 10 minutes later by an IP injection of 30 μL/g NHS. Naïve control mice received PBS only. Respiratory function was assessed at 5 hours post-injury by performing WBP. Mice were culled at 6 hours and the sera were collected to perform an ELISA and complement assay to detect the presence of anti-GM1 mAb and free human C2, respectively. (**A**) The tidal volume of control mAb mice was significantly reduced at 5 hours compared with baseline (##*P* < 0.01) and to naïve control (*P < 0.05). (**B**) The respiratory rate was elevated at 5-hours in the control mAb group compared with baseline and all other treatment groups, although not significantly. (**C**) Representative flow charts demonstrate a reduction in tidal volume with an increased respiratory rate in the control mAb group but not in the C2 inhibitor group. (**D**) Anti-GM1 mAb was detected in the sera of mice in the control mAb and C2 inhibitor groups but was absent from naïve control (*P* < 0.01). (**E**) As expected, free C2 was only detected in the control mAb group and was absent from naïve control and C2 inhibitor groups (*P* < 0.01). Results represent average ± SEM, *n* = 3 naïve control and C2 inhibitor, *n* = 4 control mAb. Each data-point represents an individual animal. A one-way ANOVA was performed to test for statistical significance.

Tidal volume and respiratory rate were assessed at 5 hours post-NHS injection and compared with baseline values (Fig. 4A and B; represented by a broken line). The tidal volume of mice treated with control mAb was significantly reduced compared with baseline and to naïve control mice. In contrast, the tidal volume of the C2 inhibitor group did not differ compared with baseline or any other treatment group (Fig. 4A). The respiratory rate was comparable between naïve control and C2 inhibitor groups; however, the respiratory rate was elevated above baseline in the control mAb group, although not significantly (Fig. 4B). These results are illustrated by the representative respiratory flow charts (Fig. 4C). These data suggest that respiratory function was rescued to comparable naïve control levels following inhibition of C2.

**Figure 5:**
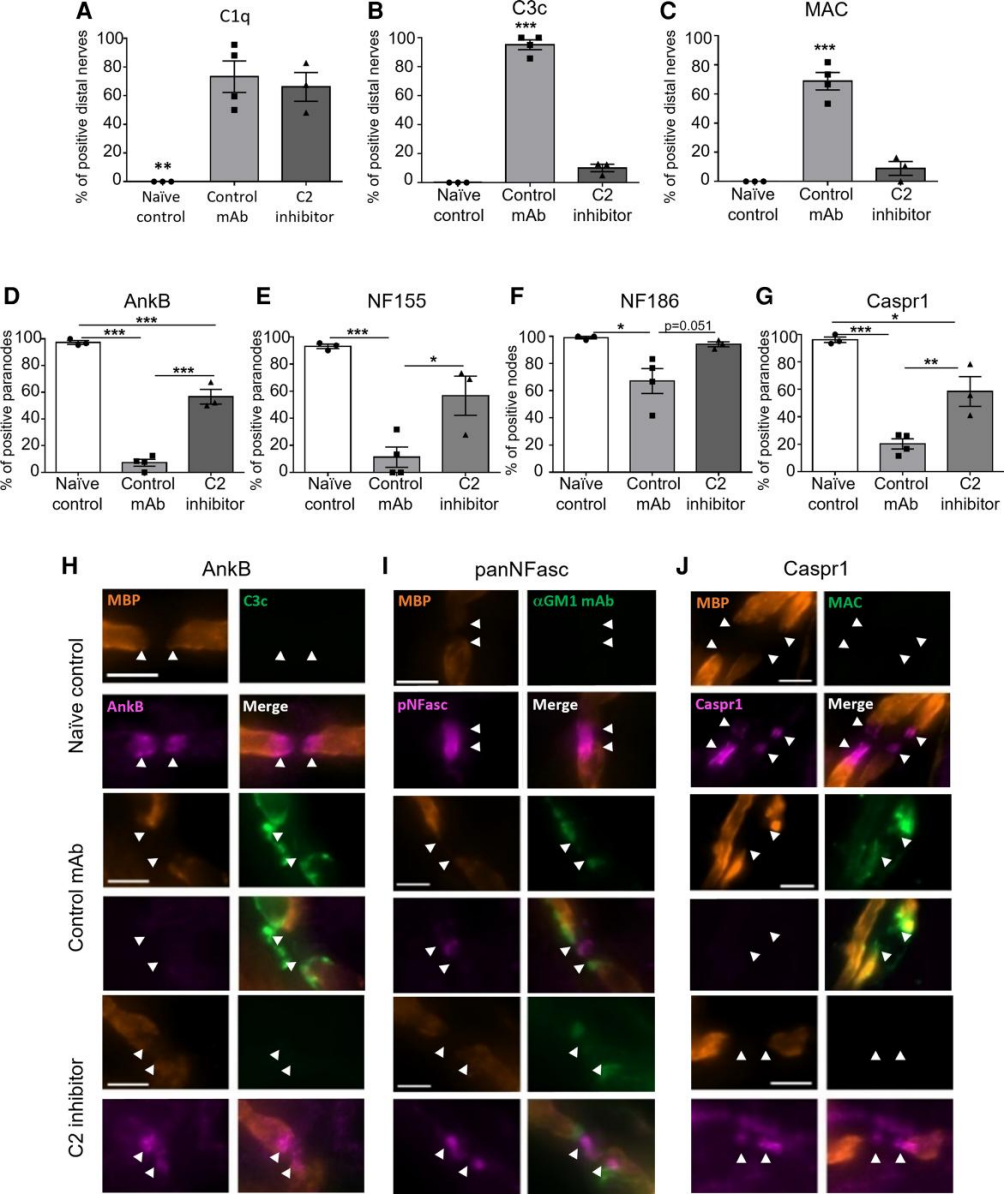
**C2 inhibition attenuates structural injury in an *in vivo* Schwann cell nodal membrane injury model.** *GalNAc-T^−/−^-Tg(glial)* mice received 50 mg/kg anti-GM1 mAb IP. The following morning, mice received an intravenous injection of 200 mg/kg of C2 inhibitor or control mAb, followed 10 minutes later with an IP injection of 30 μL/g NHS. Naïve control mice received PBS only. Mice were culled at 6 hours and the diaphragm was harvested for immunofluorescence analysis. (**A–C**) The presence of complement products, C1q, C3c and MAC at the distal nerve was assessed. (**A**) C1q deposits were detected in both the control mAb and C2 inhibitor groups (*P* < 0.01). (**B**) C3c and (**C**) MAC deposits were detected at significantly more distal nerves in the control mAb group compared with naïve control and C2 inhibitor groups (*P* < 0.001). (**D–G**) The integrity of proteins at the NoR was assessed. (**D**) AnkB was significantly reduced in the control mAb and C2 inhibitor groups compared with naïve control (*P* < 0.001). C2 inhibition offered significant protection to AnkB at the paranode in comparison to the control mAb group (*P* < 0.001). (**E–F**) A pan-NFasc antibody was used to assess paranodal NF155 and axonal NF186. (**E**) NF155 and (**F**) NF186 were significantly reduced in the control mAb group compared with naïve control and C2 inhibitor group (***P < 0.001; *P < 0.05). (**G**) CASPR1 was significantly disrupted in the control mAb (*P* < 0.001) and C2 inhibitor groups (*P* < 0.05) compared with naïve control. Treatment with the C2 inhibitor provided significant protection to CASPR1 at the paranode in comparison to the control mAb group (*P* < 0.01). **(H–J)** Representative images illustrate (**H**) AnkB, (**I**) pan-NFasc and (**J**) CASPR1 at the NoR in the naïve control and C2 inhibitor groups. In the control mAb group, anti-GM1 mAb and complement deposits are deposited at the paranodes (arrowheads) resulting in a loss of AnkB, NF155 and CASPR1 from the paranode. Scale bar = 5 μm. Results represent average ± SEM, *n* = 3 naïve control and C2 inhibitor, *n* = 4 control mAb. Each data point represents an individual animal. Statistical significance was determined by a one-way ANOVA.

An ELISA and complement assay were performed to detect the presence of anti-GM1 antibodies and levels of human free C2 in the sera of mice, respectively (Fig. 4D and E). Anti-GM1 antibody was detected in the sera of all mice in the control mAb and C2 inhibitor groups but was absent from naïve control, as expected (Fig. 4D). The results from the C2 complement assay demonstrate that human-free C2 was only detected in the sera of mice treated with control mAb and not in the naïve control group or the C2 inhibitor group (Fig. 4D), indicating the classical complement pathway was blocked at C2 in the mice treated with the C2 inhibitor. The results from the C2 complement assay indicate that one mouse from the control mAb treatment group had lower levels of C2 present in the sera; however, this mouse was not removed from analysis as each mouse in the control mAb group had comparable C3c immunofluorescence deposits detected along the distal nerve (Fig. 5B).

Diaphragm sections from the *in vivo* experiment were then assessed for the presence of complement products C1q, C3c and MAC by performing immunofluorescence analysis (Fig. 5A–C). C1q deposits were detected at distal nerves in both the control mAb and C2 inhibitor groups but not in the naïve control group, confirming the classical complement pathway had been activated by anti-GM1 mAb in these treatment groups (Fig. 5A). C3c and MAC deposits were only detected at distal nerves in the control mAb group (Fig. 5B,C, H and J). The results from the immunofluorescence analysis corroborate the complement assay results, confirming that the classical complement pathway was blocked at the C2 level in the mice treated with the C2 inhibitor.

We next investigated the integrity of cytoskeletal and axo-glial proteins at the NoR (Fig. 5D–G). Immunostaining for the glial cytoskeletal anchoring protein AnkB was severely attenuated in the control mAb group compared with all other treatment groups (Fig. 5H). Although there were significantly fewer paranodes with AnkB in the C2 inhibitor group compared with the naïve control group (Fig. 5D), C2 inhibition protected AnkB in comparison to the control mAb group. The presence of NF155 at the paranodes, which can be tethered by AnkB,^27^ was next investigated. Pan-NFasc staining was performed, which identified NF186 at the node and NF155 at the paranode (Fig. 5I). Both isoforms of neurofascin were disrupted in the control mAb group in comparison to all other treatment groups, although there was greater disruption to NF155 in the glial membrane in comparison to NF186 at the node (Fig. 5E and F). When mice were treated with the C2 inhibitor, NF186 clusters at the node were protected to comparable levels as in naïve control (Fig. 5F). In addition, the presence of NF155 at the paranode was significantly greater in the C2 inhibitor group in comparison to control mAb (Fig. 5E). To confirm that C2 inhibition protected the axo-glial junction at the paranode, the axonal binding partner of NF155, CASPR1,^28^ was assessed (Fig. 5G). Similar to AnkB and NF155 results, CASPR1 was significantly altered in the control mAb group in comparison to all other treatment groups. Blockade of the classical complement pathway at C2 significantly preserved CASPR1 staining at the paranode in comparison to control mAb (*P<* 0.01), although not when compared with naïve control (Fig. 5J).

## Discussion

Autoantibodies to glycolipids, including gangliosides, have been demonstrated to activate the complement cascade and be involved in the pathogenesis of both axonal and demyelinating variants of Guillain–Barré syndrome through serology,^7,29^ autopsy^4,30^ and animal studies.^15,19,31^ Consequently, the complement pathway has become of significant therapeutic interest, and drugs targeting C1q (involved in the initiation complex) and the C5 complement component, have now progressed through to clinical trials.^16–18^ Whilst we have established that complement inhibition attenuates injury to the axon in animal models of axonal Guillain–Barré syndrome,^15,19,31^ a beneficial role for inhibiting complement in the demyelinating variants of Guillain–Barré syndrome has yet to be established. Thus, it is currently unknown from preclinical studies whether complement inhibition would be an effective therapeutic for Guillain–Barré syndrome cases with injuries targeting the Schwann cell membranes, in comparison with AMAN cases. Importantly, this influences the outcome design and inclusion criteria for populations of patients included in clinical trials. Herein, we have demonstrated that inhibition of the early classical complement component C2, rescued the respiratory paralytic phenotype and morphological structural damage to distal motor nerve paranodes in an anti-GM1 mAb-mediated Schwann cell nodal membrane injury model. In parallel, we show that C2 inhibition attenuated injury to the distal motor nerve and nerve terminal in an established *ex vivo* AMAN model mediated by both mouse and human monoclonal anti-GM1 antibodies. These results suggest that C2 inhibition, and by inference complement component blockade at other points in the classical pathway, is likely to attenuate injury in any acute neuropathic disease mediated by complement-fixing autoantibodies.

Many complement pathway inhibitors are being developed for clinical use. The humanized antibody ARGX-117 binds to C2, thereby inhibiting the formation of the C3 convertase complex (C4bC2a).^25^ Targeting the classical complement pathway at C2 prevents the formation of anaphylatoxins C3a and C5a from downstream complement components that trigger a potent proinflammatory effect, mediating cellular chemotaxis that can cause further damage.^32^ In addition, ARGX-117 specifically inhibits the classical and lectin complement pathways, leaving the alternative complement pathway to respond to potential microbial infections.^25^ Taken together, these properties make C2 an attractive therapeutic target among the many complement components and inhibitors currently being studied.^33^ A phase 1 clinical trial is currently ongoing to evaluate the safety and efficacy of the anti-C2 mAb ARGX-117 in healthy individuals (ClinicalTrials.gov; NCT04532125). Recruitment is in progress for a phase 2 study to investigate the therapeutic benefits of complement inhibition in treating patients with multifocal motor neuropathy, a chronic condition often associated with anti-GM1 IgM antibodies (ClinicalTrials.gov; NCT05225675).

We have demonstrated that inhibiting other components of the complement pathway, like C2, offers protection to axonal integrity in our *ex vivo* mouse model of AMAN, further corroborating the involvement of the complement pathway in mediating axonal degeneration in mouse models.^15,19^ Herein, we also establish that complement inhibition prevents injury to the axon mediated by a human anti-GM1 IgM antibody cloned from a multifocal motor neuropathy patient.^34^ This has implications for not only Guillain–Barré syndrome, but other neuropathies in which autoantibodies and complement may play a key pathological role.

We predicted that injury to the glial membrane in our Schwann cell nodal membrane injury model was likely to be a result of complement activation.^20^ Here we provide evidence to indicate the involvement of the complement pathway in mediating paranodal disruption. Binding of anti-GM1 mAb to the Schwann cell membrane, particularly at the paranode, activates the classical complement pathway and the formation of MAC pores in the membrane. We know from previous studies that these MAC pores, in addition to directly affecting ionic homeostasis, are notably associated with an influx of calcium^10^ which then activates the calcium-dependent protease calpain.^12^ Many components of the underlying cytoskeleton are known calpain substrates, including actin and AnkB^13^ which are present in the cytoplasmic paranodal loops. Given the disruption observed to AnkB, it is likely that calpain is activated in the paranodes and causes subsequent disruption. One potential function of AnkB is to contribute to stabilization of the axo-glial junction by tethering glial NF155 to the glial cytoskeleton.^27^ Thus, cleavage of actin and AnkB by calpain could result in the detachment of NF155 from the glial membrane. This dispersion could in turn cause consequent mis-localization of NF155 binding partners CASPR1 and contactin on the axonal membrane.^28^ This sequence of events provides one explanation for the loss of AnkB, NF155 and CASPR1 staining in complement-disrupted paranodal membranes,^20^ as seen in tissues from the ‘control mAb-treated’ group. The extent to which Schwann cell microvilli are also targeted by anti-GM1 antibodies in this glial model remains uncertain, although it seems likely that there is some direct and/or indirect microvillal injury.^20^ The microvilli protein gliomedin participates in the clustering of NF186 at the node, maintained by a direct interaction between the two proteins.^35^ Complement-mediated injury to the glial membrane in the nodal complex would also likely disrupt Schwann cell microvilli proteins, such as gliomedin. Thus, the disturbance of NF186 from the node in our injury model could be a consequence of disruption to Schwann cell microvilli proteins. Alternatively, there is evidence to indicate that the axon is damaged secondary to injury to the glial membrane. Therefore, disruption to NF186 in our model could be early signs of secondary axonal injury. The significant disruption to the axo-glial junction at the paranode culminates in a respiratory phenotype presenting as a reduction in tidal volume and an elevation in respiratory rate; analogous to our previous findings.^15,20^ This respiratory phenotype was protected to comparable levels as naïve control by C2 inhibition.

The protocol we have developed for our *in vivo* mouse model is hyperacute, evolving over hours rather than days and thus, does not replicate the usual temporal sequence of Guillain–Barré syndrome seen in clinical practice. In patients, treatment would be invariably administered at a time point when neural tissue injury was well developed in a clinically affected subject. Intuitively, one would argue that treatment should be administered as early in the course of the clinical presentation as possible to prevent further ongoing damage caused by complement activation. Nevertheless, the data described herein provide proof of the principle that complement inhibition could offer protection to the Schwan cell membrane from AGAb-mediated injury in AIDP-like syndromes. One advantage of our injury model is that it uses the NHS as a source of complement and, in this respect, closely resembles human complement-mediated injury in patients. Previous attempts at developing an all-mouse model following passive immunization with AGAb have been unsuccessful,^36^ possibly due to variations in complement activity and regulators in laboratory mouse strains.^37^ Furthermore, the inhibitor used in this experiment is specific for human C2 and thus, the neuroprotective effects of the drug are a direct result of human complement inhibition and it would be expected to have similar effects in clinical practice. The aim of future work is to develop an extended *in vivo* injury model in *GalNAc-T^−/−^Tg(glial)* mice to provide the opportunity to administer treatment following the onset of injury, mimicking what would happen in a clinical situation.

The data from this study strongly suggest that complement is critically involved in the pathogenesis of AGAb-mediated Guillain–Barré syndrome, but it is also possible that complement-independent mechanisms are involved in Schwann cell membrane injury. Non-complement fixing autoantibodies that target the NoR are associated with chronic inflammatory demyelinating polyneuropathy.^38,39^ It is believed that these antibodies block the interaction between axonal and glial proteins that form the paranodal junction, leading to the detachment of the paranodal loops. Thus, it is possible that anti-GM1 mAb binding to paranodes could interfere with protein-protein interactions. However, evidence suggests that anti-GM1 mAb alone does not cause injury in our model.^20^ In addition, it has been demonstrated that both mouse- and patient-derived AGAbs disrupt myelination independently of complement in a myelinating co-culture system, which is considered to be due to disruption of axo-glial signalling.^40^ Nevertheless, the results presented herein indicate that injuries to the Schwann cell membrane occur via complement-dependent pathways.^20^ This supports the hypothesis that the early events in AIDP-like syndromes are the binding of complement-fixing antibodies to the surface of Schwann cell membranes, including paranodal loops.^4^ Although a universal antigenic target on peripheral nerve myelin has yet to be established in human AIDP, our *GalNAc-T^−/−^-Tg(glial)* mouse model can be used to investigate the downstream consequences of complement activation on Schwann cell membranes. In addition, this Schwann cell nodal membrane injury model may be representative of other syndromes associated with anti-GM1 antibodies including multifocal motor neuropathy;^6^ thereby supporting the use of complement inhibition as a possible treatment.^41,42^

The data presented herein provide the preclinical evidence for glial membrane protection from complement injury and thereby suggest that Guillain–Barré syndrome patients with axonal and/or Schwann cell membrane injury could reasonably be included in future complement inhibition clinical trials.

## Data Availability

The data that support the findings of this study are available from the corresponding author, upon reasonable request.

## Abbreviations

AIDP =: acute inflammatory demyelinating polyneuropathy
AMAN =: acute motor axonal neuropathy
AnkB =: ankyrin B
AGAb =: anti-ganglioside antibody
BTx =: bungarotoxin
CFP =: cyan fluorescent protein
IP =: intraperitoneally
mAb =: monoclonal antibodies
MAC =: membrane attack complex
MBP =: myelin basic protein
NF155/186 =: neurofascin-155/186
NFH =: neurofilament heavy
NHS =: normal human serum
NoR =: node of Ranvier
PBS =: phosphate buffered saline
RRID =: Research Resource Identifier
SEM =: standard error of the mean
TS =: triangularis sterni
WBP =: whole-body plethysmography
